# Influences of Probe’s Morphology for Metal Ion Detection Based on Light-Addressable Potentiometric Sensors

**DOI:** 10.3390/s16050701

**Published:** 2016-05-14

**Authors:** Chen Shao, Shuang Zhou, Xuebo Yin, Yajun Gu, Yunfang Jia

**Affiliations:** 1 College of Information Science Technology, Nankai University, No. 94 Weijin Road, Nankai District, Tianjin 300071, China; 2120140241@mail.nankai.edu.cn; 2 Department of Immunology, School of Medicine, Nankai University, No. 94 Weijin Road, Nankai District, Tianjin 300071, China; zhoushuang04@mail.nankai.edu.cn; 3 College of Chemistry, Nankai University, No. 94 Weijin Road, Nankai District, Tianjin 300071, China; xbyin@nankai.edu.cn; 4 School of Medical Laboratory, Tianjin Medical University, No. 1 Guangdong Road, Hexi District, Tianjin 300203, China; guyajun@tmu.edu.cn

**Keywords:** light addressable potentiometric sensor (LAPS), ssDNA, aptamer, morphology, metal ion

## Abstract

The sensing mechanism of binding Hg^2+^ into thymine-thymine (T-T) mismatched base pairs was introduced into a light-addressable potentiometric sensor (LAPS) with anti-Hg^2+^ aptamer as the sensing units. Three kinds of T-rich single-strand DNA (ssDNA) chains with different spacer lengths, from 0 to 12 –CH_2_ groups, were designed to investigate surface charge and morphological effects on the LAPS’ output. First, by comparing the responding of LAPS modified with three kinds of ssDNA, it was found that the best performance for Hg^2+^ sensing was exhibited by the probe without –CH_2_ groups. The detection limit of Hg^2+^ ion was 1 ppt under the optimal condition. Second, the cooperative effects of surface charge and morphology on the output were observed by the controlled experiments. The two effects were the negative charge balanced by metal cations and the morphological changing caused by the formation of T-Hg^2+^-T structure. In conclusion, not only the influences of the aptamer probe’s morphology and surface charge was investigated on the platform of LAPS, but also sensing Hg^2+^ ions was achieved for the first time by the presented aptamer LAPS.

## 1. Introduction

The light-addressable potentiometric sensor (LAPS) was one kind of silicon-based semiconductor sensor with the electrolyte-insulator-semiconductor (EIS) structure [1]. Due to its compatibility with modern integrated circuit (IC) manufacturing processes, the multi-functional LAPS principle and system have been studied in the past decades [2,3,4,5]. Accompanied with these electronic developments, LAPS was testified to undertake pH sensing [6], metal ion detection [7,8], immunoassay [9,10], cancer cell detection [11,12], and so on. It was theoretically believed that any surface electronic changes on LAPS could be expressed by LAPS’ output voltages or currents. In our previous works, it was demonstrated that grafting and hybridizing of short single-strand DNA (ssDNA) could be translated into electronic signals [13]. The DNA strand has a definitive structure and negative charge because of its sugar-phosphate backbone. The bases and surface negative charges provide the affinity toward positive metal ions. ssDNA also hybridizes to each other to form double-strand DNA (dsDNA). While ssDNA is flexible, dsDNA is rigid. All of the properties of DNA are suitable to study the effect of the surface state, including surface charge and structure, on the LAPS’ output.

Many ssDNAs have been artificially synthesized [14,15,16] and developed as the so-called aptamer sensors [17,18,19,20,21] because of the outstanding affinity and specificity to both nucleic and non-nucleic acid species. Among them, anti-Hg^2+^ ssDNA was used as a model. Oligonucleotide-based Hg^2+^ sensors provided a novel mercury-sensing strategy, which was the selective binding of mercuric ions into thymine-thymine (T-T) mismatched base pairs to form a mimic dsDNA [22,23,24,25]. The availability of the T-Hg^2+^-T scheme for mercury determination has been testified by many platforms, including fluorescence [26,27], quantum dots (QDs) luminescence [28], and dynamic light scattering [29]. Except the structural changing, the electrochemical (EC) Hg^2+^ aptasensors [22,23,25] evidenced that there was electronic transfer (ET) through both the chain of the ssDNA probe and the folded probe with a mercury-mediated hairpin structure. In-depth EC impedance spectroscopy (EIS) revealed the impedance of the ssDNA film was changed by Hg^2+^-induced self-hybridization. [30,31]. The ET signals were expressed as the currents between the working electrode (WE) and the counter-electrode (CE) and measured by the use of electro-active labels on one terminal of the mercuric probe [22,23,25], and ET-enhancing materials [25] on the electrodes or the dissociation strategy [32]. So, we thought the anti-Hg^2+^ ssDNA would be an ideal model for the electronic and morphological study of ssDNA on LAPS.

Three immobilization strategies for DNA hybridization on LAPS indicated that the highest respondse was achieved by the covalently-functionalized LAPS [33] while, by comparing this literature with other EC Hg^2+^ aptasensors [22,34], it was noticed that different lengths of carbon chains were inserted between the bases of ssDNA and the anchoring groups (–NH_2_ or –SH). The emphasis of this work was the influence of ssDNA’s morphology on the covalently-functionalized aptamer LAPS (apta-LAPS).

Here, we designed the ssDNA with different spacers of carbon link. The length of the spacer was used to study the effect of the flexible ssDNA on the LAPS’ output by the negative charge of ssDNA. In the presence of metal ions, the negative charge of the ssDNA was balanced, but the flexibility of the ssDNA was kept. This only provided the effect of surface charge on the LAPS’ output signal. However, if the metal ions were replaced with Hg^2+^, not only the surface charge was balanced, the T-Hg^2+^-T structure also increased the rigidness of the DNA strand. Therefore, the effects of the surface charge and morphology on the LAPS’ output were observed. Our work validated that a DNA strand is a powerful tool to study the effects of surface charge and morphology on the LAPS’ output. Sensing Hg^2+^ ions was also achieved with the apta-LAPS system for the first time and, to the best of our knowledge, the only research about LAPS-based mercury detection was based on the chelation effect [35]; the studies about the combination of LAPS and the T-Hg^2+^-T strategy were not found yet.

## 2. Materials and Methods

### 2.1. Chemicals and Materials

The ssDNA probes were synthesized by Sangon Biotech Co. Ltd. (Shanghai, China). The probe structure was outlined as a T-rich sensing part, cytosine (C)-rich flexible part, and anchor part. Three kinds of ssDNA probes with the same base sequences and different anchor parts were used here, named as C1, C2 and C3, given below:

C1: 5′-NH_2_-TTCTTTCTTTCCCCCCTTTGTTTGTTTGTT-3′ (1)

C2: 5′-NH_2_-(CH_2_)_6_-TTCTTTCTTTCCCCCTTTGTTTGTTTGTT-3′ (2)

C3: 5′-NH_2_-(CH_2_)_12_-TTCTTTCTTTCCCCCCTTTGTTTGTTTGTT-3′ (3)

T- and C-rich parts were the core of ssDNA probes which were designed symmetrically with T-rich parts on both ends and six C bases in the center. Furthermore, in the left and right T-rich parts, C and guanine (G) bases are intersected, respectively, as well as complimentarily and symmetrically, as described in Equations (1)–(3). The amino group (NH_2_–) is used as an anchor element to graft ssDNA (5′) probes on chips. Probes (C1, C2, and C3) have three kinds of anchor parts used here, which used NH_2_– to anchor to the core part directly (C1), through 6 and 12 –CH_2_ groups (C1 and C2, respectively).

Reagents are listed here: (1) (3-aminopropyl) triethoxysilane (APTES) was purchased from Sigma-Aldrich Co. Ltd. (Shanghai, China); (2) glutaraldehyde (GA) was purchased from Alfa Aesar Co. Ltd., Ward Hill, MA, USA; (3) mercury standard sample was purchased from National Center of Analysis and Testing for Nonferrous Metals and Electronic Materials (Beijing, China); (4) deionized (DI) water (18.25 MΩ·cm) was used during the experiments. All other chemicals (HCl, phosphate, acetone, Tris-acetate, *etc.*) were of analytical grade.

The reagents in this work are as follows: (1) APTES solution was prepared by diluting in DI water with the volume ratio (v/v) of 1:10, and adjusted to pH 7.4; (2) the concentration of GA dilution solution was 2.5% (v/v); (3) Tris-acetate solution was prepared by diluting Tris (hydroxymethyl) in DI water and adjusting pH to 7.4 by acetate, its ultimate concentration was 50 mM; (4) 100 mM PBS was prepared by diluting Na_2_HPO_4_ and NaH_2_PO_4_ in DI water and adjusting pH to 7.4 by HCl; (5) the mercury standard sample solution was diluted to 0.001, 0.01, 0.1, 1, 10, 100 ppb, in 50 mM Tris-acetate (pH 7.4), and shaking gently; (6) the aptamer probes were diluted in 100 mM PBS (pH 7.4) separately, the final concentrations of them being 1 μM.

### 2.2. Preparation of the LAPS Chip

**Figure 1:**
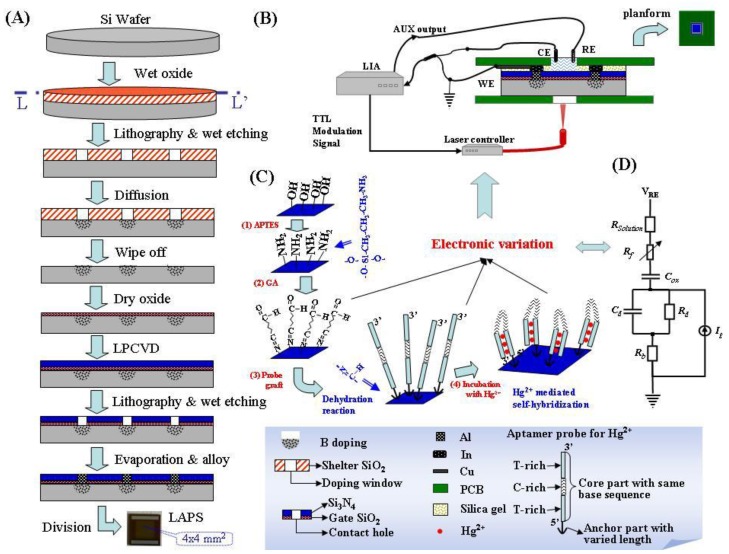
Illustration of an aptamer LAPS system (**A**) fabricating process of LAPS; (**B**) the encapsulated LAPS chips and the detection system; (**C**) the protocol for ssDNA probe immobilization and mercuric ion formed self-hybridization on LAPS; and (**D**) the equivalent circuit for aptamer LAPS.

The progress of preparing LAPS was described in Figure 1A. The naked LAPS chips were fabricated on the substrate of p-type silicon wafer with crystal face <111>, electric resistivity of 8–12 Ω·cm. There is the highest planar atom density in crystal face <111> than in the other normally-used crystal faces (<110> and <100>). This means, by using the <111> substrate, more photo-excited carriers per unit area could be generated when LAPS is illuminated from the backside, as depicted in Figure 1B. The whole steps were outlined here. By wet oxidation, a layer of silicon oxide (SiO_2_) with the thickness of 700~800 nm was grown; the doping window, which was depicted in Figure 1A, was patterned by lithography and wet etching, the remains were used as the shelter of the following boron diffusion. After wiping off this shelter SiO_2_, dry oxidation and low pressure chemical vapor deposition (LPCVD) were executed to grow a layer of gate SiO_2_ (50~70 nm) and nitride silicon (200–300 nm). Contacting holes were etched through these two layers and terminated at the Si wafer. Electronic contacts were made by filling these holes with evaporated Al and alloying. After thinning from the backside to about 200–300 µm, LAPS chips were divided. The naked chips were encapsulated by a sandwiched structure as presented in Figure 1B. The hole on the backside was designed for intercepting the illumination, the top hole was for executing chemical tests. The top view of encapsulated chip was given as an inset on the top right corner. The depressed center on it was used as a container for liquid analyte.

### 2.3. LAPS Detection System

The LAPS detection block was constructed according to the three-electrode EC method, in which Ag/AgCl was used as the reference electrode (RE), Pt was used as CE, and LAPS was WE. According to LAPS’ sensing mechanism [1], RE voltage (*V*_REF_) was controlled to maintain the LAPS working state in tests, and LAPS’ signal was the current between CE and WE, which was transferred to voltage (*V*_out_) by the lock-in amplifier (LIA, SR830 from Stanford Research Systems Inc., Sunnyvale, CA, USA). The illumination source is the laser controller, LOS-BLD_0980-600m-C, from Hi-tech Opto-electronic Co. Ltd, Beijing, China. The wavelength of illumination was selected as 980 nm and its modulation frequencies could be controlled by the auxiliary TTL output of LIA.

### 2.4. Surface Modification and LAPS Measurements

The modification steps are depicted schematically in Figure 1C and their experimental conditions described here. Each of the operating steps was executed in the depressed center of encapsulated LAPS (Figure 1B), and followed by rising with DI water. The LAPS chips were cleaned by Piranha solution, firstly, then the following steps were executed, which were outlined here: (1) 50 μL of APTES solution, at 50 °C for 2 h; (2) 50 μL GA solution, at room temperature (20 °C) for 1 h; (3) 50 μL probe solution, at 4 °C overnight; activating the ssDNA probe is performed by 100 mM PBS (pH 7.4) at 4 °C for 24 h; (4) incubation with mercuric solutions of different concentrations from low to high (0.001–100 ppb), at room temperature for 30 min. Measurements were executed after steps (2)–(4) by the presented LAPS detection system in Figure 1B.

The surface of LAPS after each modification step was characterized by XPS, the details about XPS measurements, results and discussions were presented in the supplementary materials.

## 3. Results and Discussion

### 3.1. Electronic Characteristics of BLANK LAPS

Based on the LAPS principle, one of three working states, which are accumulation, depletion, and inversion, may be formed at the interface of SiO_2_ and Si, depending on the charges and the effective bias voltage on SiO_2_ [1]. For the p-type Si substrate used here, the accumulation state was formed when there were negative charges or voltage; the depletion state was formed when positive charges or voltage were applied, while too much positive charge or voltage would cause inversion. In this section, electronic characteristics of BLANK LAPS were measured and discussed to choose an appropriate working condition for the following experiments.

As mentioned above, the photo-generated electron-hole pairs diffusing from bulk wafer were separated by the induced electric field at the interface of SiO_2_/Si. For the LAPS used in this paper, if it was in the depletion state, holes were scanned into depleted region and electrons were dispelled into the bulk wafer. It was very similar to the charging phenomenon in the depletion layer of a PN junction. Thus, this part was equivalent to a capacitor *C*d and a resistor *R*d, as depicted in Figure 1D. The effective values of them were changed by the sensing process which was modeled as the resistor of the ssDNA film (*R*f) at the controlled bias voltage and modulation frequency. Meanwhile, if the illumination was modulated with a specified frequency, there would be an alternating current (AC) with the same frequency. In the detection system of Figure 1B, the modulated AC was transformed to the output voltage (*V*_out_).

**Figure 2:**
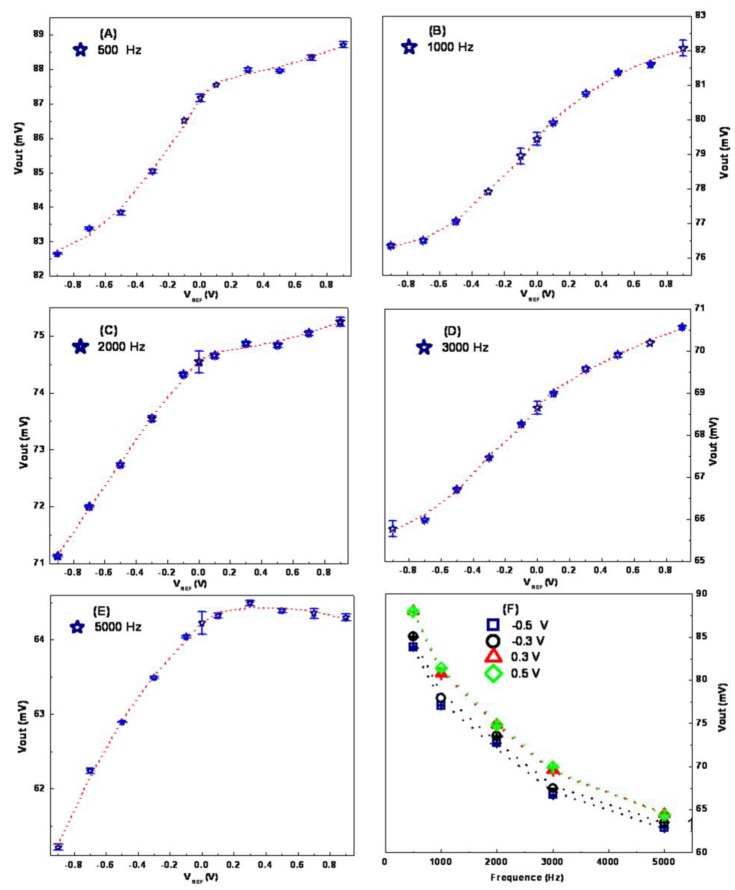
Basic electronic features of LAPS when the illumination was modulated with frequency (**A**) 500 Hz, (**B**) 1000 Hz, (**C**) 2000 Hz, (**D**) 3000 Hz and (**E**) 5000 Hz. (**F**) The influences of the illumination’s modulating frequencies were examined.

The measured data were plotted in the curves of *V*_out_ *vs.* the reference voltage (*V*_REF_), presented in Figure 2. Here *V*_REF_ was limited between −0.9 V and 0.9 V because metals in RE and CE would be dissolved if it was larger than 1 V. At the same time, modulation frequencies of illumination were maintained at 500, 1000, 2000, 3000, and 5000 Hz, respectively. In these controlled working conditions, *V*_out_ was measured and the electronic feature curves were presented in Figure 2A–E. It was found there were two regions in the curves: the linear region and the saturation region. For example, in Figure 2B when the values of *V*_REF_ are lower than −0.5 V or larger than 0.5 V, the curve’s slope becomes smaller. In addition, when the modulation frequency was changed from 500 to 5000 Hz, the curves’ shapes changed little, in general. However, it was found by comparing the curves in Figure 2A–E, at the same bias voltage, that *V*_out_ was changed by the modulation frequency, as shown in Figure 2F.

For the application of our detector, the control of *V*_REF_ was needed in order to maintain LAPS working in the linear region. Since this linear region is related to the depleted interface state, which was mentioned in the first paragraph of this section. At the constant positive *V*_REF_, holes were dispelled from the depletion layer for the p-Si used here, the electronic variations caused by sensing film would change *R*f, which would change the effective bias voltage applied to the insulator (Si_3_N_4_ film). Then, the amplitudes of the electronic field, *C*_d_ and *R*_d_ will be modified by the sensing process; as a consequence, *V*_out_ will be altered. That means at the same working condition (bias voltage and modulation frequency), the target molecule-induced electronic changes on LAPS could be distinguished by *V*_out_, while for other states, like the accumulation state, carriers’ concentrations at the interface of Si-SiO_2_ are higher than in the bulk of the Si wafer; it does not facilitate the diffusion and separation of photo-generated carriers, so LAPS’ output voltage is lower than the depletion state. In fact, there exists a third state, called inversion, but in this system bias voltage is limited, so it is not tested.

Based on the discussion given above, working conditions in the following steps were chosen as follows: bias voltages were ±0.5 V and ±0.3 V, modulation frequencies were 500, 750, and 1000 Hz, respectively.

### 3.2. Influences of Grafted Probes on LAPS

**Figure 3:**
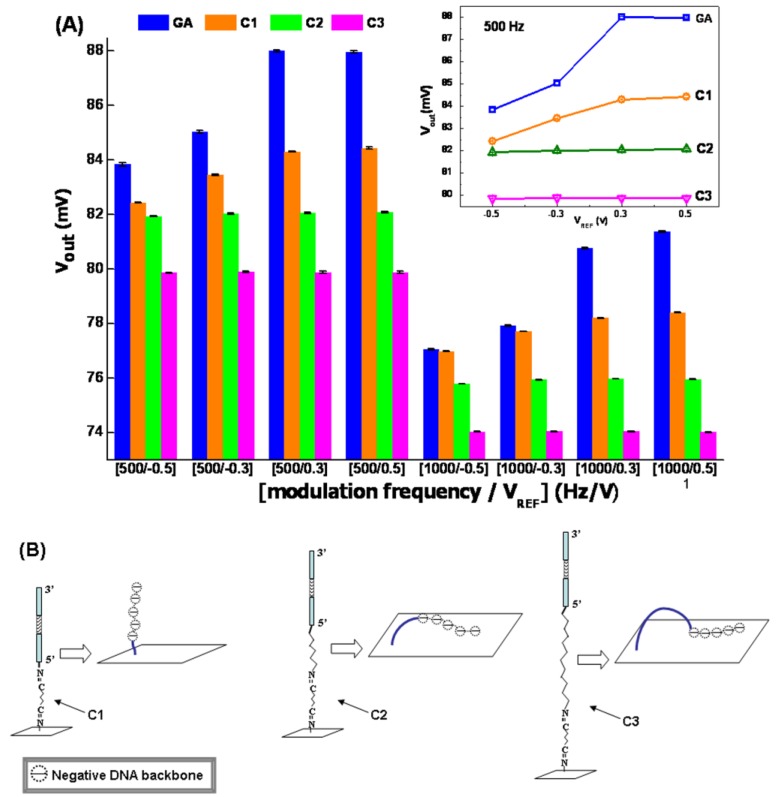
Influence of grafting ssDNA probes with different flexibility on LAPS and the schematic interpretation for the experimental results. (**A**) LAPS’ output voltages’ variation after binding with ssDNA probes at different working conditions which was denoted as [modulation frequency/*V*_REF_], while the electronic features of aptamer-modified LAPS at the selected modulation frequency were analyzed (given in the inset); and (**B**) the shapes of three probes C1, C2, and C3 on the LAPS surface.

The influences of grafted ssDNA probes with same sequences and different lengths of anchor parts (as presented in Figure 1 and Equations (1)–(3)) were tested at the eight selected working conditions, and the results were presented in Figure 3A. It could be found that, though both the base sequence in the probes Ci (i = 1, 2, 3) and the working condition were same, different LAPS output voltages were measured. As shown on Figure 3A, the columns of Ci (i = 1, 2, 3) samples were lower than GA ones. Furthermore, the inset in Figure 3A indicated that electronic feature curves became flatter with the probes’ immobilization, as well as the increasing of the carbon chain length. For C2 and C3 samples, there was almost no linear region in their curves. We thought this phenomenon was the synthetic result of the electric resistance along the probes (*R*f), negative charge of the DNA backbone, and the flexibility of these probes.

First, with the immobilization of probes, extra impedance was induced by ssDNA [30,31], there would be extra electric consumption on it, so the effective bias voltage applied on the interface of SiO_2_ and Si would be reduced though the working condition was maintained. That is to say, the effective bias voltage on GA-treated LAPS would be higher than ssDNA-grafted ones. According to the basic electronic features in Figure 2, it was equivalent with reducing *V*_REF_. Then, the lowered columns in Figure 3A for C1, C2, and C3 were reasonable.

Second, the LAPS surface would be negatively charged by the ssDNA phosphoric acid skeleton if the ssDNA probes lay down, as depicted in Figure 3B. The explanation is proposed here. The Hg^2+^ sensing ssDNA sequence was grafted on the LAPS surface by the reaction of aldimine condensation. Since varied anchor parts were designed in probes (C1, C2, C3), the flexibility was enhanced from C1 to C3 with the increasing carbon chain length. This facilitated the bending of ssDNA probes and enabled the negative charges (possessed by the phosphoric acid skeleton) to be absorbed on the LAPS surface. As a consequence, the depleted state which was generated by the selected *V*_REF_ would be weakened by the absorbed negative charges. Then, the lower *V*_out_ could be measured. The more flexible the probe, the more charges were absorbed, as illustrated in Figure 3B, and the more weakened was the depleted state. Thus, at the same working conditions, the columns’ height in Figure 3A descended from C1 to C3.

Third, it was deduced that the quantities of absorbed negative charges on C2 and C3 samples were so large that the selected *V*_REF_ could not make LAPS work in the depleted state, so the curves of C2 and C3 were not in the linear region.

### 3.3. Hg^2+^ Detection by Aptamer LAPS

**Figure 4:**
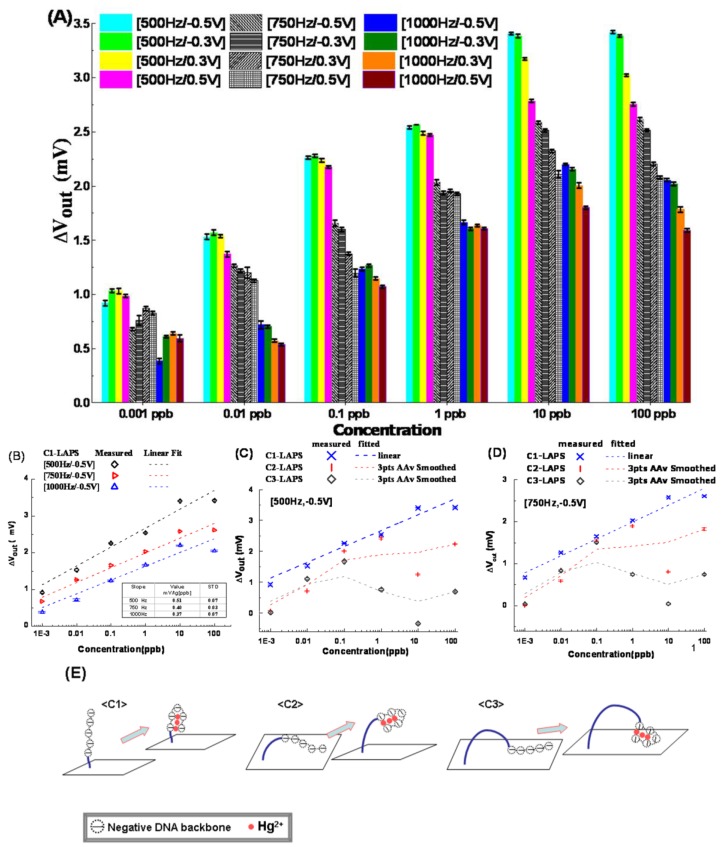
Results and explanations for anti-Hg^2+^ modified LAPS response to Hg^2+^. (**A**) Histogram of C1 modified LAPS (C1-LAPS) response to different concentration of Hg^2+^ solutions at twelve working conditions; (**B**) linear fitted curves of C1-LAPS for sensitivity analyzing; (**C**,**D**) at optimal working condition, which was [500 Hz, −0.5 V] and [750 Hz, −0.5 V], the response curves of Ci-LAPS (i = 1, 2, 3) were compared in the same semi-log coordinate; and (**E**) morphological changes of the three probes in the forming of T-Hg^2+^-T structure were depicted to analyze the experimental results.

The ssDNA-modified sensors named as Ci-LAPS (i = 1, 2, 3) were used for the Hg^2+^ detection in this section. The responses of C1-LAPS to different concentrations of Hg^2+^ were examined at the selected working conditions. The shift of output voltages (*V*_out_) after incubation with different concentrations of Hg^2+^ agents were plotted in Figure 4. It could be found that the responding concentration of C1-LAPS was as low as 0.001 ppb and *V*_out_ increased with the increasing Hg^2+^ concentration.

We thought it could be explained by the ET and impedance changing of the ssDNA [22,23,24,30,31]. Though the exact mechanism ET along the ssDNA was still under debate, it was accepted that there was electrical conduction through DNA molecules. In this experiment, the existence of Hg^2+^ made the ssDNA chain folded and form a so-called T-Hg^2+^-T hairpin structure, as depicted in Figure 4E. Then, the length of the C1 chain was shortened and the cross-section area of the folded chain was doubled, both of which were in favor of increasing ET and the electronic conductance, so the value of *R*f could be reduced. According to the discussion proposed in Section 3.2, at the constant working condition, the effective bias voltage would be increased by the reducing of *R*f, so the positive *V*_out_ could be measured. Furthermore, higher Hg^2+^ concentration would generate more folded C1 on the LAPS surface, and the enhanced columns along the horizontal axis for each constant working condition could be understood.

Second, the curves of *V*_out_ *vs.* the concentration Hg^2+^, when *V*_REF_ was −0.5 V, were plotted in the semi-log coordinates as shown in Figure 4B. It was found that the measured *V*_out_ grew in a linear manner. The fitted lines were generated by the method of least squares in Origin^®^ (Nankai University, Tianjin, China). It was indicated that at the selected modulation frequencies, the largest slope of the linear fitted curves was 0.514 mV/lg[ppb] with a standard deviation (STD) of 6.8% when the working condition was 500 Hz and −0.5 V. At the same time, the lowest STD was 3.9% with a slope of 0.33 mV/lg[ppb] when the working condition was 750 Hz and −0.5 V.

Third, the response characteristics of C2- and C3-modified LAPS (C2-LAPS and C3-LAPS) were also examined and compared with C1-LAPS when *V*_REF_ was −0.5 V, and modulation frequencies were 500 and 750 Hz, respectively. The measured data points, fitted lines, and smoothed curves were presented in Figure 4C,D. It was found there was a close to linear response when concentrations were less than 1 ppb and 0.1 ppb for C2-LAPS and C3-LAPS, respectively. When concentrations were increased, both C2-LAPS and C3-LAPS curves fluctuated up and down. The explanation for the measured results could be diagrammed by Figure 4E and discussed here.

Since the core parts of C2 and C3 were identical to C1, the T-Hg^2+^-T hairpin structure could also be formed on C2-LAPS and C3-LAPS. At this time, with the folding of ssDNA, not only ssDNA resistance, but also its flexibility, was reduced. The prostrate probes on C2-LAPS and C3-LAPS would stand up as illustrated in Figure 4E. The upright probes reduced the negative charges on the LAPS surface, which would be helpful to increase *V*_out_. Thus, according to the discussions about the effects of ssDNA immobilization on LAPS in Section 3.2 and the respondse of C1-LAPS in this section, these two effects explained the positive increasing *V*_out_ when Hg^2+^ concentrations were below 1 ppb for C2-LAPS and 0.1 ppb for C3-LAPS.

The change of the distance between negative charges of DNA and the surface is considered in the case of C2- and C3-LAPS, because the probes in the case of C2- and C3-LAPS were more flexible than in the case of C1-LAPS. The influence of probes’ falling down and standing up happened in the case of C2- and C3-LAPS, as shown in the Figure 3B and Figure 4E. However, this phenomena did not happen in the case of C1-LAPS, since the state of the probes in C1-LAPS was upright at all time, as depicted in the Figure 3B and Figure 4E. Thus, the change of the distance between negative charges of DNA and the surface is not considered in the discussion for the sensitivity of C1-LAPS.

The sensitivity of C1-LAPS was better than C2-LAPS and C3-LAPS, as it could respond to as low as 0.001 ppb, but C2-LAPS and C-LAPS could not, as given in Figure 4C,D. We thought it was because the amounts of negative charges induced by falling ssDNA on C2-LAPS and C3-LAPS were large enough that only a small amount of upstanding probes and their folding could not change the LAPS working state. For the concentration 0.001 ppb, C2-LAPS and C3-LAPS could not work in the depleted state, so they could not exhibit the sensibility as C1-LAPS did when the concentration was as low as 0.001 ppb. Compared with the detection limit of [25], we admit that their detection limit is better than ours. However, in our detection method, there is no need for additional material like graphene or nano-Au, so it will be easier to operate.

At last, for the observed fluctuation in Figure 4C,D, it might be the combined influence of the electrostatic adsorption and drooping of the folded ssDNA probe on C2-LAPS and C3-LAPS (depicted in Figure 4E). On one hand, according to the discussion in Section 3.3, LAPS surfaces were negatively charged by the grafted probes Ci (i = 1, 2, 3) and the sequence of their charging degree could be C3-LAPS > C2-LAPS > C1-LAPS. Then, the electrostatic forces to attract cations (like Hg^2+^ or H_3_O^+^) in analyte would be decreased in this order. These unspecific cations made LAPS surface positively charged so *V*_out_ was increased (according to the discussion in Section 3.2). On the other hand, the flexible carbon chains on C2 and C3 probes could not support the folded core parts, so they could not stand on LAPS as upright as C1, making the LAPS surface negatively charged and reducing *V*_out_. Based on these two opposite effects, it was suspected that, on C2-LAPS and C3-LAPS, there might be some competition between them which resulted in the fluctuation of *V*_out_. Since there was no flexible part in the C1, it was thought that both the charging effect and the unspecific adsorption on C1-LAPS were less than C2-LAPS and C3-LAPS.

### 3.4. Response of Apta-LAPS to Other Metal Ions

**Figure 5:**
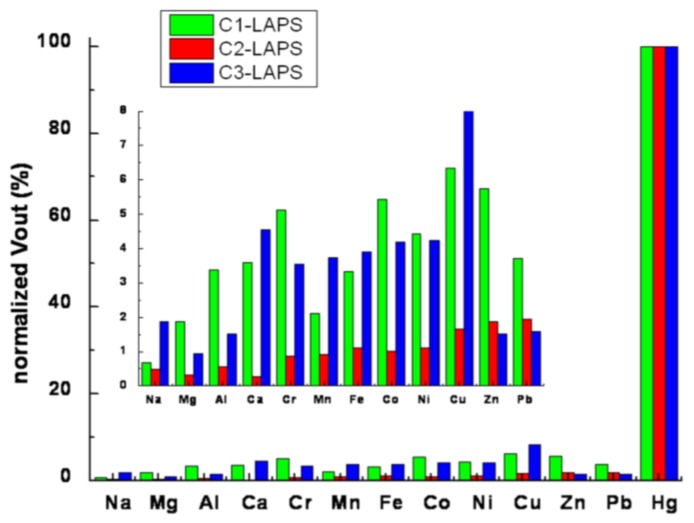
The response of Ci-LAPS (i = 1, 2, 3) to other metal ions and the comparison with Hg^2+^.

Under the working conditions of *V*_REF_ = −0.5 V and modulation frequency 750 Hz, the responding signals of Ci-LAPS (i = 1, 2, 3) to other metal ions (Na, Mg, Al, Ca, Cr, Mn, Fe, Co, Ni, Cu, Zn, and Pb) were also examined and compared with Hg^2+^, as given in Figure 5. The concentrations of other metal ions and Hg^2+^ solutions used in this experiment were 1 ppb.

The *V*_out_ of Ci-LAPS (i = 1, 2, 3) from the metal ions were normalized according to the of deviation standardization method. The other metal ions showed a more weakened response than Hg^2+^. The electrostatic interaction was the main reason for the response to the other metal ions. The adsorbed cations have two effects: (1) increasing the positive level of the effective bias voltage on SiO_2_ which increased *V*_out_ according to Figure 3; (2) the cations’ adsorption was different from the force of Hg^2+^ with the ssDNA probes on LAPS, as these captured cations on LAPS could not change the pattern of the probes on the substrate. Thus, though there were responses for other metal ions, they were relatively small.

Furthermore, we found the trend that the heavy metals’ responses were higher. We thought that there might be coordination linkages formed since there were unoccupied orbitals on the metal ions and lone pairs of electrons on the probes.

## 4. Conclusions

Based on the LAPS platform, the influences of ssDNA probes’ flexibility and surface charges were measured and analyzed. According to the results and discussions mentioned above, it was demonstrated that T-Hg^2+^-T direct electronic sensing could be realized by LAPS. Though surface charge changing and morphological changing both existed on LAPS, the latter overwhelmed the former. Moreover, it was also indicated that the increased flexibility of ssDNA probes would have a negative effect on the responding feature. As a consequence, it was suggested that apta-LAPS’, as the combination of LAPS and ssDNA, morphological sensing mechanism was the key point in constructing similar aptamer sensors.

## Supplementary Materials

The following are available online at www.mdpi.com/1424-8220/16/5/701/s1, Figure S1: Surface characterization of LAPS: (**A**) core spectra and the fitted curves of C1s; (**B**) N1s, (**C**) Si2p, and (**D**) O1s after being cleaned by Piranha solution (named as BLANK), modified by APTES and GA.

## References

[1] Hafeman D.G., Parce J.W., McConnell H.M. (1988). Light-addressable potentiometric sensor for biochemical systems. Science.

[2] Ermolenko Y.E., Yoshinobu T., Mourzina Y.G. (2004). Laser-scanned silicon transducer (LSST) as a multisensor system. Sens. Actuators B Chem..

[3] Schoning M.J., Wanger T., Wang C., Otto R., Yoshinobu T. (2005). Development of a handheld 16 channel pen-type LAPS for electrochemical sensing. Sens. Actuators B Chem..

[4] Yoshinobu T., Schoning M.J., Otto R., Furuichi K., Mourzina Y., Ermolenko Y., Iwasaki H. (2003). Portable light-addressable potentiometric sensor (LAPS) for multisensor applications. Sens. Actuators B Chem..

[5] Zhang Q., Wang P., Parak W.J., George M., Zhang G. (2001). A novel design of multi-light LAPS based on digital compensation of frequency domain. Sens. Actuators B Chem..

[6] Sawada K., Mimura S., Tomita K., Nakanishi T., Tanabe H., Ishida M., Ando T. (1999). Novel CCD-based pH imaging sensor. IEEE Trans. Electron Devices.

[7] Ha D., Hu N., Wu C.X., Kirsanov D., Legin A., Khaydukova M., Wang P. (2012). Novel structured light-addressable potentiometric sensor array based on PVC membrane for determination of heavy metals. Sens. Actuators B Chem..

[8] Wan H., Ha D., Zhang W., Zhao H., Wang X., Sun Q., Wang P. (2014). Design of a novel hybrid sensor with microelectrode array and LAPS for heavy metal determination using multivariate nonlinear calibration. Sens. Actuators B Chem..

[9] Gu L.B., Han J.H., Qui D.F., Zhang H. (2005). The Research of Light Addressable Potentiometric Sensor on Detection of Alpha Feto-protein. Chin. J. Anal. Chem..

[10] Jia Y.F., Gao C.Y., He J., Feng D.F., Xing K.L., Wu M., Liu Y., Cai W.S., Feng X.Z. (2012). Unlabeled multi tumor marker detection system based on bioinitiated light addressable potentiometric sensor. Analyst.

[11] Gu Y., Ju C., Li Y., Shang Z., Wu Y., Jia Y., Niu Y. (2015). Detection of circulating tumor cells in prostate cancer based on carboxylated graphene oxide modified light addressable potentiometric sensor. Biosens. Bioelectron..

[12] Jia Y., Qin M., Zhang H., Niu W., Li X., Wang L., Li X., Bai Y., Cao Y., Feng X. (2007). Label-free biosensor: A novel phage-modified Light Addressable Potentiometric Sensor system for cancer cell monitoring. Biosens. Bioelectron..

[13] Jia Y., Yin X.B., Zhang J., Zhou S., Song M., Xing K.L. (2012). Graphene oxide modified light addressable potentiometric sensor and its application for ssDNA monitoring. Analyst.

[14] Tuerk C., Gold L. (1990). Systematic evolution of ligands by exponential enrichment: RNA ligands to bacteriophage T4 DNA polymerase. Science.

[15] Ellington A.D., Szostak J.W. (1990). *In vitro* selection of RNA molecules that bind specific ligands. Nature.

[16] Miyake Y., Togashi H., Tashiro M., Yamaguchi H., Oda S., Kudo M., Tanaka Y., Kondo Y., Sawa R., Fujimoto T. (2006). Mercury^II^-Mediated Formation of Thymine-Hg^II^-Thymine Base Pairs in DNA Duplexes. J. Am. Chem. Soc..

[17] Tombelli S., Minunni M., Mascini M. (2005). Analytical applications of aptamers. Biosens. Bioelectron..

[18] Lu M., Xu L., Zhang X., Xiao R., Wang Y. (2015). Ag(I)-coordinated hairpin DNA for homogenous electronic monitoring of hepatitis C virus accompanying isothermal cycling signal amplification strategy. Biosens. Bioelectron..

[19] O’Sullivan C.K. (2002). Aptasensors-the future of biosensing?. Anal. Bioanal. Chem..

[20] Song S., Wang L., Li J., Fan C., Zhao J. (2008). Aptamer-based biosensors. Trend. Anal. Chem..

[21] Kandimalla V.B., Ju H. (2004). New Horizons with A Multi Dimensional Tool for Applications in Analytical Chemistry—Aptamer. Anal. Lett..

[22] Han D., Kim Y.R., Oh J.W., Kim T.H., Mahajan R.K., Kim J.S., Kim H. (2009). A regenerative electrochemical sensor based on oligonucleotide for the selective determination of mercury (II). Analyst.

[23] Kong R.M., Zhang X.B., Zhang L.L., Jin X.Y., Huan S.Y., Shen G.L., Yu R.Q. (2009). An ultrasensitive electrochemical “turn-on” label-free biosensor for Hg^2+^ with AuNP-functionalized reporter DNA as a signal amplifier. Chem. Comm..

[24] Ono A., Togashi H. (2004). Highly Selective Oligonucleotide-Based Sensor for Mercury (II) in Aqueous Solutions. Angew. Chem. Int. Ed..

[25] Zhang Y., Zeng G.M., Tang L., Chen J., Zhu Y., Xiao X.H., He Y. (2015). Electrochemical Sensor Based on Electrodeposited Graphene-Au Modified Electrode and NanoAu Carrier Amplified Signal Strategy for Attomolar Mercury Detection. Anal. Chem..

[26] Xu J.P., Song Z.G., Fang Y., Mei J., Jia L., Qin A.J., Sun J.Z., Ji J., Tang B.Z. (2010). Label-free fluorescence detection of mercury (II) and glutathione based on Hg^2+^-DNA complexes stimulating aggregation-induced emission of a tetraphenylethene derivative. Analyst.

[27] Tan D., He Y., Xing X., Zhao Y., Tang H., Pang D. (2013). Aptamer functionalized gold nanoparticles based fluorescent probe for the detection of mercury (II) ion in aqueous solution. Talanta.

[28] Xie W.Y., Huang W.T., Luo H.Q., Li N.B. (2012). CTAB-capped Mn-doped ZnS quantum dots and label-free aptamer for room-temperature phosphorescence detection of mercury ions. Analyst.

[29] Ma L.N., Liu D.J., Wang Z.X. (2014). Gold Nanoparticle-Based Dynamic Light Scattering Assay for Mercury Ion Detection. Chin. J. Anal. Chem..

[30] Liu S., Kang M., Yan F., Peng D., Yang Y., He L., Wang M., Fang S., Zhang Z. (2015). Electrochemical DNA Biosensor Based on Microspheres of Cuprous Oxide and Nano-chitosan for Hg(II) Detection. Electrochim. Acta.

[31] Lin Z., Li X., Kraatz H.B. (2011). Impedimetric Immobilized DNA-Based Sensor for Simultaneous Detection of Pb^2+^, Ag^+^, and Hg^2+^. Anal. Chem..

[32] Liu X., Sun C., Wu H., Zhang Y., Jiang J., Shen G., Yu R. (2010). Label-Free Electrochemical Biosensor of Mercury Ions Based on DNA Strand Displacement by Thymine-Hg(II)-Thymine Complex. Electroanalysis.

[33] Bronder T., Wu C.S., Poghossian A., Werner C.F., Keusgen M., Schöning M.J. (2014). Label-free Detection of DNA Hybridization with Light-addressable Potentiometric Sensors: Comparison of Various DNA-immobilization Strategies. Proced. Eng..

[34] Tang X., Liu H., Zou B., Tian D., Huang H. (2012). A fishnet electrochemical Hg^2+^ sensing strategy based on gold nanoparticle-bioconjugate and thymine-Hg^2+^-thymine coordination chemistry. Analyst.

[35] Li Y., Zhang Z., Cai W., Gao X.M., Wang P. (2010). Mercury ion selective light addressable potentiometric sensor based on PVC membrane. J. Zhejiang. Univ. Sci. B.

