# Supplementary material for: Influences of Probe’s Morphology for Metal Ion Detection Based on Light-Addressable Potentiometric Sensors
Source: Sensors (Basel). 2016 May 14;16(5):701. doi: 10.3390/s16050701 (PMC4883392; doi:10.3390/s16050701)
Supplement: Supplementary file 1 [file sensors-16-00701-s001.doc]

Supplementary Materials: Influences of Probe’s Morphology for Metal Ion Detection Based on Light-Addressable Potentiometric Sensors

Chen Shao, Shuang Zhou, Xuebo Yin, Yajun Gu and Yunfang Jia

The XPS characterizations for LAPS surfaces before and after being modified by APTES and GA were performed on an XPS apparatus (PHI-5300, PerkinElmer, Waltham, MA, USA) employing a monochromatic Mg Ka radiation source (1253.6 eV). The survey scan range was 0–1100 eV and the electron take-off angle was fixed at 45°. The energy resolution of the analyzer was 0.8 eV, and the sensitivity was 80–1600 KCPS. The peaks in the elemental core-level spectra were fitted using UNIX on an Apollo Domain series 3500.

XPS measurements were performed to evaluate the modification effect of animation and aldehydization. The LAPS chips after being cleaned by Piranha solution, animated by APTES, and treated by GA were named as BLANK, APTES, and GA, respectively. The core spectra of their main elements (C1s, N1s, Si2p, and O1s) are presented in Figure S1A–D.

| 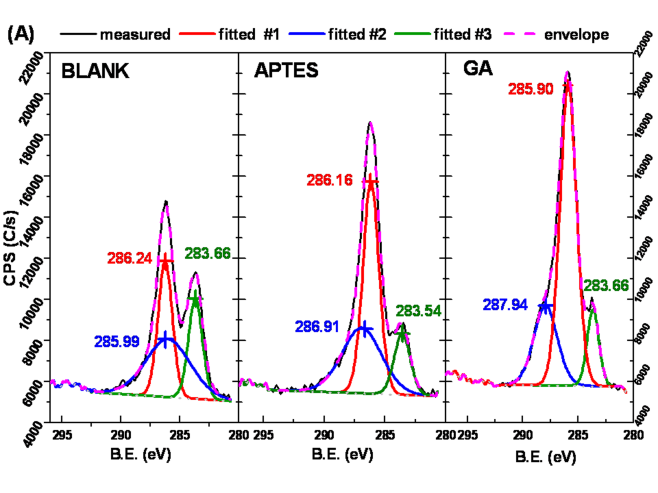 | 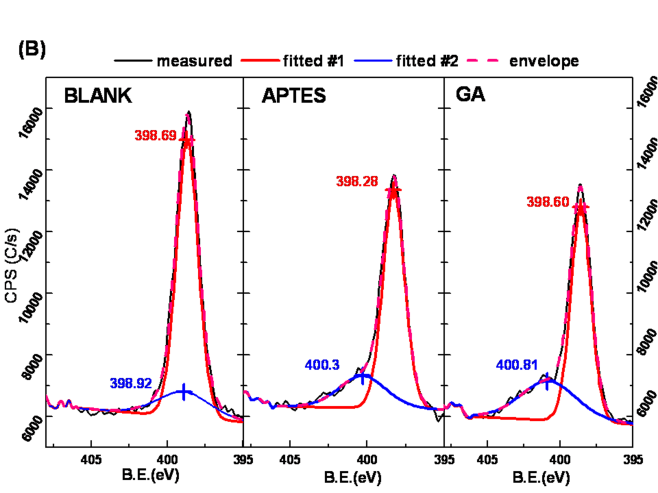 |
| --- | --- |
| 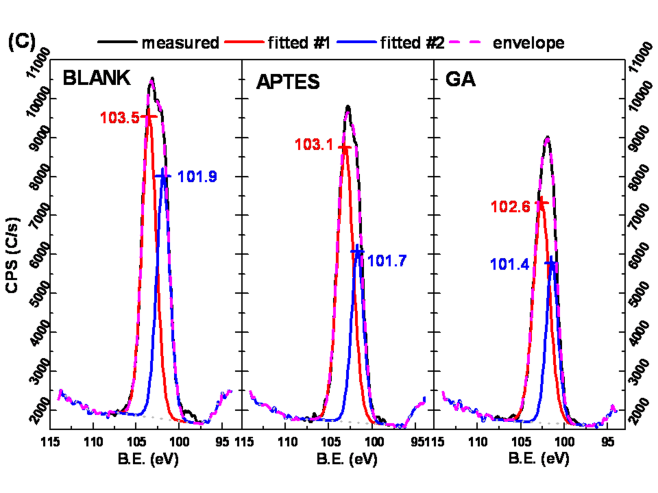 | 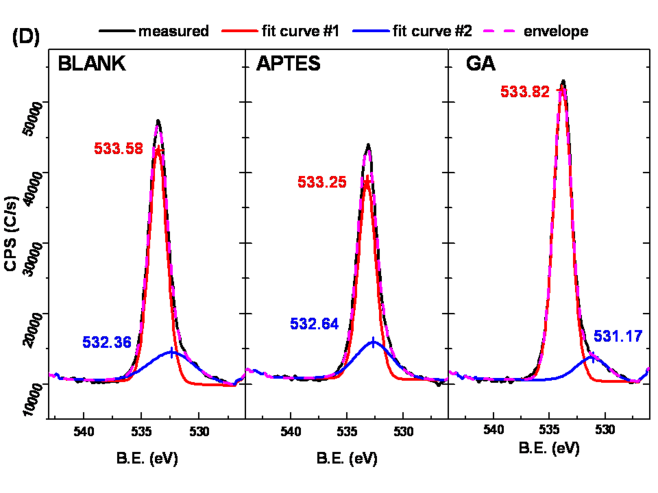 |

**Figure S1.** Surface characterization of LAPS: (**A**) core spectra and the fitted curves of C1s; (**B**) N1s, (**C**) Si2p, and (**D**) O1s after being cleaned by Piranha solution (named as BLANK), modified by APTES and GA.

Firstly, the increased intensities of C1s-core spectra indicated that more carbon-containing materials were deposited on LAPS. It was in accordance with the reagents used here, which were APTES and GA. Both of them were organic materials and had carbon in their molecules. While we thought the existence of carbon elements in the BLANK sample was brought about by contamination. The fitted curves, which were labeled as fitted #1–3 in Figure S1A, evidenced that the main bonding types of C1s in BLANK and APTES are C–C (283~284 eV) and C–O (285~286 eV). With the modification by GA, the double bond which possessed higher bind energy (BE), was brought into the GA sample, which was the blue peak of GA at 287.94 eV in Figure S1A. This was in agreement with our expected double bonds, C=N and C–O as illustrated in Figure S1C.

Secondly, the core spectra of N1s and Si2p were analyzed to clarify the states of Si and N on BLANK sample, as well as to confirm the forming of amine and C=N groups on APTES and GA samples. For the pure (not oxide) Si_3_N_4_, BEs of Si2p and N1s were about 101.5 eV and 397.1 eV, respectively, but the fitted curves of BLANK’s N1s in Figure S1B peaked at higher levels, about 398.69 eV and 398.92 eV. The peaks of fitted curves for BLANK’s Si2p in Figure S1C were 101.9 eV and 103.5 eV. It was believed that the emergence of higher BEs in both N1s and Si2p were caused by the oxidation. This meant the groups of –OH on cleaned BLANK LAPS were reasonable, as depicted in Figure S1C.

At the same time, there were few changes in the Si2p core spectra of APTES and GA samples (Figure S1C), except for the lowering of the peaks’ intensities which were caused by covering of APTES and GA. While, for the N1s core spectra, higher BEs (400.3 eV and 400.81 eV) were found in APTES and GA than BLANK (Figure S1B), it was deduced that these BEs belonged to the amine groups and C=N groups, as given in Figure S1C.

Finally, the appearance of aldehyde groups (O=C–H) on GA-modified LAPS was evidenced by comparing fitted curves of BLANK, APTES, and GA samples’ O1s core spectra. The peak of fitted curve #2 in the spectrum of GA was located at 531.17 eV, which is the BE of O1s in aldehyde groups. This was coincident with C1s spectra.
